# NKG2A is a late immune checkpoint on CD8 T cells and marks repeated stimulation and cell division

**DOI:** 10.1002/ijc.33859

**Published:** 2021-11-10

**Authors:** Linda Borst, Marjolein Sluijter, Gregor Sturm, Pornpimol Charoentong, Saskia J. Santegoets, Mandy van Gulijk, Marit J. van Elsas, Christianne Groeneveldt, Nadine van Montfoort, Francesca Finotello, Zlatko Trajanoski, Szymon M. Kiełbasa, Sjoerd H. van der Burg, Thorbald van Hall

**Affiliations:** ^1^ Department of Medical Oncology Oncode Institute, Leiden University Medical Center Leiden The Netherlands; ^2^ Institute of Bioinformatics, Innsbruck Medical University Innsbruck Austria; ^3^ Department of Medical Oncology, National Center for Tumor Diseases University Hospital Heidelberg, Applied Tumor Immunity, German Cancer Research Center (DKFZ) Heidelberg Germany; ^4^ Department of Pulmonology Erasmus Medical Center Rotterdam The Netherlands; ^5^ Department of Biomedical Data Sciences Leiden University Medical Center Leiden The Netherlands

**Keywords:** CD8 T cells, immune checkpoint, NKG2A, TGF‐β, tumor immunity

## Abstract

The surface inhibitory receptor NKG2A forms heterodimers with the invariant CD94 chain and is expressed on a subset of activated CD8 T cells. As antibodies to block NKG2A are currently tested in several efficacy trials for different tumor indications, it is important to characterize the NKG2A^+^ CD8 T cell population in the context of other inhibitory receptors. Here we used a well‐controlled culture system to study the kinetics of inhibitory receptor expression. Naïve mouse CD8 T cells were synchronously and repeatedly activated by artificial antigen presenting cells in the presence of the homeostatic cytokine IL‐7. The results revealed NKG2A as a late inhibitory receptor, expressed after repeated cognate antigen stimulations. In contrast, the expression of PD‐1, TIGIT and LAG‐3 was rapidly induced, hours after first contact and subsequently down regulated during each resting phase. This late, but stable expression kinetics of NKG2A was most similar to that of TIM‐3 and CD39. Importantly, single‐cell transcriptomics of human tumor‐infiltrating lymphocytes (TILs) showed indeed that these receptors were often coexpressed by the same CD8 T cell cluster. Furthermore, NKG2A expression was associated with cell division and was promoted by TGF‐β in vitro, although TGF‐β signaling was not necessary in a mouse tumor model in vivo. In summary, our data show that PD‐1 reflects recent TCR triggering, but that NKG2A is induced after repeated antigen stimulations and represents a late inhibitory receptor. Together with TIM‐3 and CD39, NKG2A might thus mark actively dividing tumor‐specific TILs.

## Graphical abstract

What's new?

The immune inhibitory receptor NKG2A is of emerging interest in cancer immunotherapy, with antibodies that target and interrupt NKG2A, in combination with PD‐L1 blockade, yielding promising antitumor responses in ongoing clinical trials. NKG2A expression on CD8 T cells, however, has not been fully charted and thus little is known about its regulation. Here, in vitro analyses of the kinetics of inhibitory receptor expression identify NKG2A as a late immune checkpoint. Its expression, induced upon repeated antigen encounter, is frequently associated with TIM3 and CD39. The findings suggest that NKG2A is a potential marker for the identification of tumor‐specific T cells.

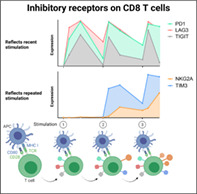

**Abbreviations**
CTLA‐4: cytotoxic T‐lymphocyte associated protein 4
HLA: human leukocyte antigen
HPV: human papillomavirus
LAG3: lymphocyte activation gene 3
NK: natural killer
NKG2A: natural killer group 2 member A
NKG2D: natural killer group 2 member D
PD‐1: programmed death 1
PD‐L1: programmed death ligand 1
TCR: T cell receptor
TGF‐β: transforming growth factor
TIGIT: T‐cell immunoglobulin and ITIM domain
TIL: tumor infiltrating lymphocyte
TIM‐3: T cell immunoglobulin domain and mucin domain 3

## 1 INTRODUCTION

The unprecedented clinical impact of immune checkpoint blockade therapy introduced a new era of cancer treatment. Thus far, antibodies to inhibitory receptors cytotoxic T‐lymphocyte associated protein 4 (CTLA‐4) and the programmed death 1 (PD‐1)/programmed death ligand 1 (PD‐L1) axis have demonstrated clinical responses, including complete regression of lesions, and are approved for a dozen of tumor types.^1^, ^2^, ^3^ The mere presence of such inhibitory receptors on T cells witnesses the importance of a proper balance in the activation status of these cells and the need for negative feedback during a physiological immune response.^4^ Inhibitory receptors dampen T cell activation via different mechanisms: distraction from stimulatory receptors by competition with shared ligands (CTLA‐4, TIGIT), inhibition of the direct T cell receptor (TCR) signaling modules (PD‐1, TIM‐3), restraining metabolic changes (PD‐1), alterations at the transcriptional level (PD‐1), interfering with proliferation (lymphocyte activation gene 3 [LAG‐3]) and suppressing inflammatory cues (CD39, CD73).^1^, ^5^, ^6^, ^7^, ^8^, ^9^, ^10^ The impact of CTLA‐4 and PD‐1 on homeostasis of T cell immunity is evident from the autoimmune pathologies that develop in their absence. CTLA‐4‐deficient mice develop lymphoproliferative disease within a few months, leading to severe myocarditis and pancreatitis.^11^ Similarly, mice deficient for PD‐1 demonstrate lupus‐like proliferative arthritis and glomerulonephritis.^12^ This emphasizes the crucial role for inhibitory receptors to maintain peripheral tolerance, to regulate the extend of immune responses at the site of inflammation and balance costimulatory receptors carrying immunoreceptor tyrosine‐based activation motifs (ITAMs).^13^ Most inhibitory receptors might not operate as an on/off switch but rather orchestrate local immune responses in collaboration with activation signals in the micromilieu where they are expressed, as this differs between tissues, cell types and differentiation state of the cells.^4^

Recently, the immune inhibitory receptor natural killer group 2 member A (NKG2A) drew attention as a new checkpoint molecule for immunotherapy of cancer.^14^, ^15^, ^16^, ^17^, ^18^ Interruption of the NKG2A receptor with its ligand human leukocyte antigen (HLA)‐E improved antitumor responses of PD‐L1 blockade therapy and therapeutic peptide vaccination in more than five different mouse tumor models.^15^, ^18^ Moreover, several ongoing clinical trials show promising responses when the NKG2A antibody monalizumab is combined with PD‐L1 blockade.^19^ Though NKG2A is constitutively expressed as a dimer with CD94 on a majority of mature natural killer (NK) cells, CD8 T cells require TCR‐mediated activation before NKG2A/CD94 is displayed and frequencies of NKG2A^+^ CD8 T cells are increased in tumor tissues.^14^, ^18^, ^20^ The regulation of NKG2A expression on CD8 T cells is only sparsely described and more insight is necessary on its regulation, on the CD8 T cell subsets that upregulate NKG2A, and even on frequencies of NKG2A^+^ CD8 T cells in different human tumor types. In our current study, we demonstrate that NKG2A is a late inhibitory receptor expressed upon repeated cognate antigen stimulation, in contrast to inhibitory receptors PD‐1, TIGIT and LAG‐3, which are immediately expressed upon the first stimulation. These expression kinetics were revealed in well‐controlled synchronized culture systems using naïve mouse CD8 T cells and confirmed in single‐cell transcriptomics of human cancer tumor‐infiltrating lymphocytes (TILs). Our data indicate that NKG2A marks proliferating tumor‐specific TILs.

## 2 MATERIALS AND METHODS

### 2.1 Single‐Cell RNA sequencing of TIL from human cancers

#### 2.1.1 Head and neck squamous cell carcinoma

In order to analyze coexpression of inhibitory receptors in human TIL, 13 treatment‐naïve patients with oropharyngeal squamous cell carcinoma (OPSCC) were included in our study as part of an observational study focusing on circulating and local immune responses in head and neck cancer (P07.112).^21^ Patients were included when histopathology confirmed the presence of a carcinoma and after signing informed consent. All patients received standard‐of‐care treatment. Human papillomavirus (HPV) typing and p16ink4a‐IHC were performed on formalin‐fixed paraffin‐embedded material as described previously.^21^ Live, CD3^+^ T cells and CD56^+^ NK cells were isolated from thawed single‐cell tumor samples using the dead cell removal kit (Miltenyi Biotec), followed by combined CD3/CD56‐guided magnetic cell sorting using CD3 and CD56 microbeads (Miltenyi Biotec) according manufacturer's instructions. Postisolation viability was more than 70%. Single‐cell sequencing was performed as described previously by us.^21^ In brief, between 2108 and 6107 sorted cells per sample were loaded on a Chromium Single Cell Controller (10x Genomics), lysed and barcoded. Reverse transcription of polyadenylated mRNA from single cells was performed inside each gel bead emulsion using the 5′ gene expression pipeline. Next‐generation sequencing libraries were prepared in a single bulk reaction, and transcripts were sequenced using the HiSeq4000 System (Illumina). Single‐cell transcriptome FASTQ files were processed with cell ranger v3.0.0 (10x Genomics) using the GRCh38 reference genome. Scanpy^22^ was used for quality control and downstream analysis. In brief, we removed cells with <2000 counts, <700 detected genes, or >11% mitochondrial reads based on the distribution of the quality metrics. Cells with more than one TCR‐β or more than two TCR‐α chains were identified using scirpy^23^ and discarded as putative doublets, in addition to computational doublet detection using solo.^24^ Ribosomal, mitochondrial and T‐cell receptor genes were excluded from downstream analysis. Counts were normalized per cell and log‐transformed. The 6000 most highly variable genes were selected and used for unsupervised Leiden‐clustering.^25^ Embeddings were visualized using UMAP.^26^ Cell‐types were annotated based on the leiden clusters and cell‐type specific marker genes. The T‐ and NK cell compartment was extracted and subjected to subclustering. Differential gene expression analysis was performed using edgeR,^27^ including the number of detected genes into the linear model. A Nextflow pipeline^28^ to reproduce the single‐cell analysis, data analysis reports, including the full list of differentially expressed genes is available from https://icbi-lab.github.io/borst2021. All software dependencies are packaged as Singularity containers. The sequencing coverage and quality statistics for each sample are summarized in Table S1.

#### 2.1.2 Nonsmall cell lung carcinoma, hepatocellular carcinoma, colorectal carcinoma and breast carcinoma

Previously published datasets of single‐cell transcriptome results were used for reproduction and visualization. Data for 14 nonsmall cell lung carcinomas^29^ (9055T cells from blood and tumor), 5 hepatocellular carcinomas^30^ (4070T cells from blood and tumor) and 12 colorectal carcinomas^31^ (11138T cells from peripheral blood, adjacent normal and tumor tissues) can be found at: http://lung.cancer-pku.cn, http://hcc.cancer-pku.cn and http://crctcell.cancer-pku.cn/, respectively. The code to reproduce the analyses and to generate the figure for two breast carcinomas (5759T cells from blood and tumor) was kindly shared by Dr Peter Savas.^32^ Quantified gene counts for single‐cell sequencing are available at the Gene Expression Omnibus (GEO) under accession number GSE110686.

### 2.2 Mice

C57BL/6 mice were purchased from Charles River Laboratories (L'Arbresle, France). Ovalbumin‐specific T cell receptor (TCR)‐transgenic OT‐I mice (stock #: 003831) recognizing the H‐2K^b^‐restricted SIINFEKL epitope and gp100‐specific TCR‐transgenic pmel mice (stock #: 005023) recognizing the H‐2D^b^‐restricted EGSRNQDWL epitope were obtained from The Jackson Laboratory and bred to express the congenic marker CD45.1 (Ly5.1) (B6.SJL‐CD45.1). The conditional transforming growth factor (TGF)‐β receptor II knockout mice (TβRII^fl/fl^)^33^ were kindly obtained from Dr P. ten Dijke (Leiden, the Netherlands) and were crossed with CD8a‐driven Cre‐knock‐in mice (Jackson Laboratories strain 008766) to generate CD8Cre^+/−^TβRII^fl/fl^ (CD8 TGF‐βRII KO) and CD8Cre^−/−^TβRII^fl/fl^ (TGF‐βRII WT) mice. Genomic PCR was conducted to analyze the genotypes of mice using ear DNA and gene‐specific primers for the conditional TGF‐βRII locus^33^ and Cre construct (CRE transgene 5′‐CAA TGG AAG GAA GTC GTG GT‐3′; wt 5′‐CAC ACA TGC AAG TCT AAA TCA GG‐3′; CRE common 5′‐TGG GAT TTA CAG GGC ATA CTG‐3′). All mice were housed in individually ventilated cages, maintained under specific pathogen‐free conditions and used at 6 to 20 weeks of age.

### 2.3 Cell lines

The dendritic cell line D1 is a long‐term growth factor‐dependent immature splenic DC line derived from C57BL/6 (H‐2^b^) mice and was obtained from Dr P. Ricciardi‐Castagnoli and cultured as described.^34^ Generation of the engineered APC cell line SAMBOK (MEC.B7.SigOVA) was described before.^35^ SAMBOK is a mouse embryonic fibroblast clone transfected with CD80 and the minigene SigOVA, encoding the OVA_257‐264_ SIINFEKL epitope behind a signal peptide for efficient loading onto H‐2K^b^ and was obtained from Dr S. Schoenberger. To create SAMBOK.Qa‐1^b^ cells, the codon‐optimized gene of Qa‐1^b^ (H‐2T23 gene ENSMUST00000102678.4) (IDT integrated DNA technologies) was transduced using lentiviral pCDH vector (System bioscience, CD500.1). Qa‐1^b^ expressing SAMBOK cells were selected by flow cytometry cell sorting. The tumor cell line TC‐1 (RRID:CVCL_4699) expresses the HPV16‐derived oncogenes E6 and E7 and activated Ras oncogene and was a gift from T.C. Wu (John Hopkins University, Baltimore, USA). The B16F10 melanoma cell line (RRID:CVCL_0159) was purchased from the American Type Culture Collection (ATCC‐CRL6475). The MC38 cell line (RRID:CVCL_B288) is a chemically induced colon adenocarcinoma and was obtained from Dr Ossendorp. Pancreatic cancer cell lines KPC2 and KPC3 (RRID:CVCL_A9ZK) were derived from primary tumors of KPC mice on C57BL/6 background and obtained from Dr Balkwill.^36^ The AE17.OVA mesothelioma line (RRID:CVC_LJ85) was isolated from C57BL/6J mice injected with asbestos fibers and transfected with cDNA encoding for secretory OVA and kindly provided by Prof Delia J. Nelson (Curtin University, Perth, Australia). To authenticate TC‐1, B16F10, KPC and MC38 cell lines, STR DNA profiling was performed with Mouse CellsCheck protocol (IDEXX). D1 and SAMBOK cells have no deviating STR profiles as they are genetically normal. All experiments were performed with mycoplasma‐free cells as tested with PCR. Cells of low passage number were used for all experiments. Cell lines were cultured in Iscove's modified Dulbecco's medium (IMDM; Invitrogen) supplemented with 8% FCS (Gibco), 2 mM L‐glutamine (Life Technologies), 50 IU/mL penicillin (Life Technologies) and 50 μg/mL streptomycin (Life Technologies), except for AE17.OVA, which was cultured in RPMI1640 medium containing L‐glutamine supplemented with Glutamax, 25 mmol/L HEPES, 50 mg/mL gentamicin (all obtained from Gibco) and 5% fetal bovine serum (Capricorn Scientific). All cell lines were cultured in a humidified atmosphere and at 5% CO_2_.

### 2.4 Flow cytometry on mouse tumor and spleen samples

For tumor cell inoculation of AE17.OVA cells, mice were i.p. injected with 0.3 × 10^6^cells in 300 μL PBS. TC‐1 (1 × 10^5^), KPC2 and KPC3 (2 × 10^5^), MC‐38 (1 × 10^5^) or B16F10 (1 × 10^5^) tumor cells were s.c. injected in 200 μL PBS/0.1% BSA in the flanks of mice. For analysis of tumor‐infiltrating populations, mice were sacrificed at indicated time points and tumors were harvested, disrupted in small pieces, and incubated with Liberase TL (Roche) in IMDM for 15 minutes at 37°C. Single‐cell suspensions were prepared by mincing the tumors through a 70‐μm cell strainer (BD Biosciences). Cells were first incubated with Zombie Aqua Fixable Viability Kit (Biolegend, cat. 423102) in PBS for 20 minutes at room temperature. Subsequently, cells were washed and resuspended in staining buffer (PBS + 0.5% BSA + 0.05% sodium azide) supplemented with 2.4G2 Fc block for 15 minutes on ice. Cells were then incubated with monoclonal antibodies for 30 minutes on ice. Antibodies used were against CD3 (145‐2C11), NK1.1 (PK136) from BD, CD4 (RM4‐5), TIM‐3 (RMT3‐23) from Biolegend, CD45.2 (104), CD8a (53‐6.7), NKG2A (16a11), CD94 (18d3) PD‐1 (RMP1‐30) and LAG‐3 (eBioC9B7W) from eBioscience. Staining of Qa‐1^b^ was performed in a 2‐step procedure with biotin‐labeled anti‐Qa‐1^b^ (clone 6A8.6F10.1A6) from BD followed by streptavidin‐APC from eBioscience. Samples were acquired on a BD Fortessa flow cytometer, and results were analyzed using the FlowJo software (TreeStar).

### 2.5 In vitro CD8 T cell coculture

CD8 T cells were isolated from spleen and lymph nodes of pmel mice or OT‐I mice using mouse CD8 T lymphocyte enrichment set based on negative selection (BD Biosciences; Cat# 558471). For in vitro stimulation of the CD8 T cells from pmel mice, D1 cells were harvested using 2 mM EDTA (suspension and adherent cells) and matured by adding 5 μg/mL LPS for 20 to 24 hours. The matured D1 cells (2 × 10^5^) were seeded in wells of a 24‐well plate and loaded for 1 to 2 hours with 1 μg/mL short EGP peptide. Pmel CD8 T cells were then added (1 × 10^6^/well). Plates were centrifuged for 1 minute at 1000 rpm to initiate cell contacts and cocultured for 20 to 24 hours after which loosely attached cells were transferred to fresh 24‐wells plates at a concentration of 0.5 × 10^6^ cells/well in the presence of recombinant mouse cytokines IL‐7 (2 ng/mL; R&D Systems) and IL‐15 (Peprotech) and/or human recombinant TGF‐β1 (Peprotech) at the indicated concentrations. For the in vitro stimulation of the CD8 T cells from OT‐I mice, 2 × 10^5^ cells SAMBOK cells were seeded per well in 24‐well plates and incubated for 1 to 2 hours. Then OT‐I cells were added (1 × 10^6^/well) and plates were centrifuged for 1 minute at 1000 rpm to initiate cell contacts. After 20 to 24 hours of coculture the nonadherent OT‐I cells were gently harvested, washed and transferred to fresh 24‐wells plates at a concentration of 0.5 × 10^6^ cells/well in the presence of aforementioned cytokines. Every 3 to 4 days cells were split and cytokines and medium refreshed or harvested for subsequent stimulation.

### 2.6 Flow cytometry on cocultured CD8 T cells

Cells were first incubated with Zombie Aqua Fixable Viability Kit (Biolegend, cat. 423102) in PBS for 20 minutes at room temperature. Subsequently, cells were washed and incubated with monoclonal antibodies for 30 minutes on ice. Antibodies used were against CD8a (53‐6.7), CD44 (IM‐7), CD62L (MEL‐14), CD94 (18d3), NKG2A (16a11), PD‐1 (RMP1‐30), LAG‐3 (eBioC9B7W), TIM‐3 (RMT3‐23), TIGIT (1G9), CD226 (DNAM‐1; TX42.1) and natural killer group 2 member D (NKG2D) (CX5) from BD, BioLegend or eBioscience. Staining of CD244 was performed in a two‐step procedure with biotin‐labeled anti‐CD244 (2B4) followed by streptavidin‐BV605. Samples were acquired on a BD Fortessa flow cytometer and the Aurora 3‐ and 5‐laser (Cytek Biosciences). Results were analyzed using the FlowJo software (TreeStar). Cell sorting was performed on a BD FACSAria II flow cytometer (BD Biosciences).

### 2.7 RNAseq on OT‐I T cells

CD8 T cells from OT‐I mice were cultured according to the coculture method mentioned above at Day 0, 7 and 14 and isolated for cell sorting at Day 22 at which they were sorted for NKG2A expression. RNA from the NKG2A positive and the NKG2A negative fractions were isolated by NucleoSpin RNA XS (Machery‐Nagel) and quality confirmed using the Agilent 2100 bioanalyzer with the RNA 6000 Nano LabChip kit. RNA‐seq library prep was performed using the Smart‐seq2 protocol + Nextera XT. Sequencing of the prepared libraries was executed on the NovaSeq 6000 PE150bp. Processing of the FASTQ files was performed using the BioWDL RNA‐seq pipeline (https://github.com/biowdl/RNA-seq) version v1.0.0. FastQC (version 0.11.7) and MultiQC (version 1.7) were used to assess the quality of the reads and processed results. Cutadapt (version 2.4) with default settings was used for adapter clipping and quality trimming. The processed reads were aligned to the mouse reference genome GRCm38 (mm10) using STAR (version 2.6.0c) with default settings. A raw read count matrix was generated using HTSeq (version 0.9.1) and available at https://www.ebi.ac.uk/ena/browser/submit under study identifier PRJEB47938 or ERP132259. Ensembl gene annotation version 96 was used for expression quantification. The sequencing coverage and quality statistics for each sample are summarized in Table S2.

### 2.8 Statistical analysis

Details on statistics used can be found in figure legends. All statistical analyses were performed using GraphPad Prism software version 8 (GraphPad). In all cases a *P*‐value of .05 and below was considered significant (*), *P* < .01(**), *P* < .001 (***) and *P* < .0001 (****) as highly significant.

## 3 RESULTS

### 3.1 NKG2A expression is frequently coexpressed with PD‐1 in the tumor microenvironment

**FIGURE 1:**
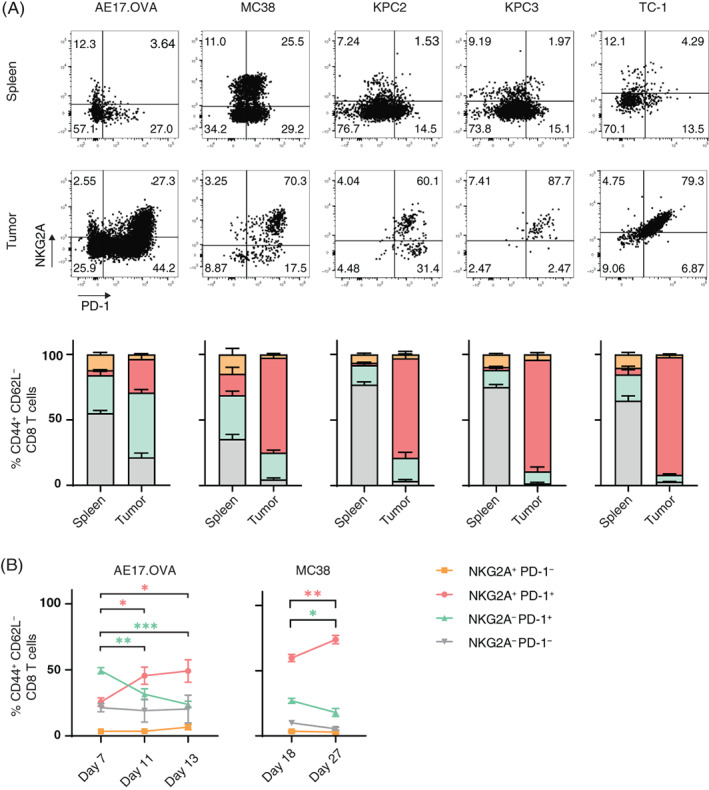
The fraction of NKG2A‐positive CD8 TIL increases over time. (A) Representative flow cytometry plots and quantification of CD8 T cells expressing NKG2A and PD‐1. Tumors and spleens of untreated C57BL/6 mice were harvested, dispersed and stained for flow cytometry. Frequencies of activated CD8 T cells (CD44^+^CD62L^−^) expressing NKG2A and/or PD‐1 are depicted from AE17.OVA mesothelioma (n = 6), MC38 colon carcinoma (n = 6), two independent KPC pancreas carcinomas (n = 5) and HPV16‐induced TC‐1 tumors (n = 5). Proportion of CD3^+^CD8^+^ T cells with the CD44^+^CD62L^−^ phenotype was much higher in tumors than in spleens (Figure S5). Data presented are means ± SEM. (B) Dynamics of CD8 T cell subsets in AE17.OVA (n = 6) and MC38 (n = 6) tumor models. Four subsets were plotted over time. One‐way ANOVA with Holm‐Sidak's multiple comparisons test was used for statistical analysis [Color figure can be viewed at wileyonlinelibrary.com]

To analyze expression of NKG2A and compare this to the well‐studied checkpoint PD‐1, we isolated tumors and spleens from groups of untreated mice bearing five different mouse tumor types (Figures 1 and S1). Expression of NKG2A and PD‐1 were determined on activated effector CD8 T cells (CD44^+^CD62L^−^) from these two sites using flow cytometry. Higher proportions of checkpoint‐positive CD8 T cells were found in tumors compared to spleens (Figure 1A). In nearly all tumor types the vast majority of activated CD8 T cells in the tumor were positive for NKG2A and PD‐1. In the AE17.OVA mesothelioma, however, a fraction of PD‐1 positive CD8 T cells were negative for NKG2A (Figure 1A). Interestingly, the PD‐1^+^ proportion that gained NKG2A increased over time in this model, as well as in the MC38 colon carcinoma model (Figure 1B). This effect did not relate to tumor size as such and was less observed in the spleen (Figure S1C,D), suggesting that NKG2A expression is induced after repeated TCR‐mediated activation of CD8 T cells within the tumor.

### 3.2 NKG2A surface expression is induced after repeated TCR‐mediated stimulation

**FIGURE 2:**
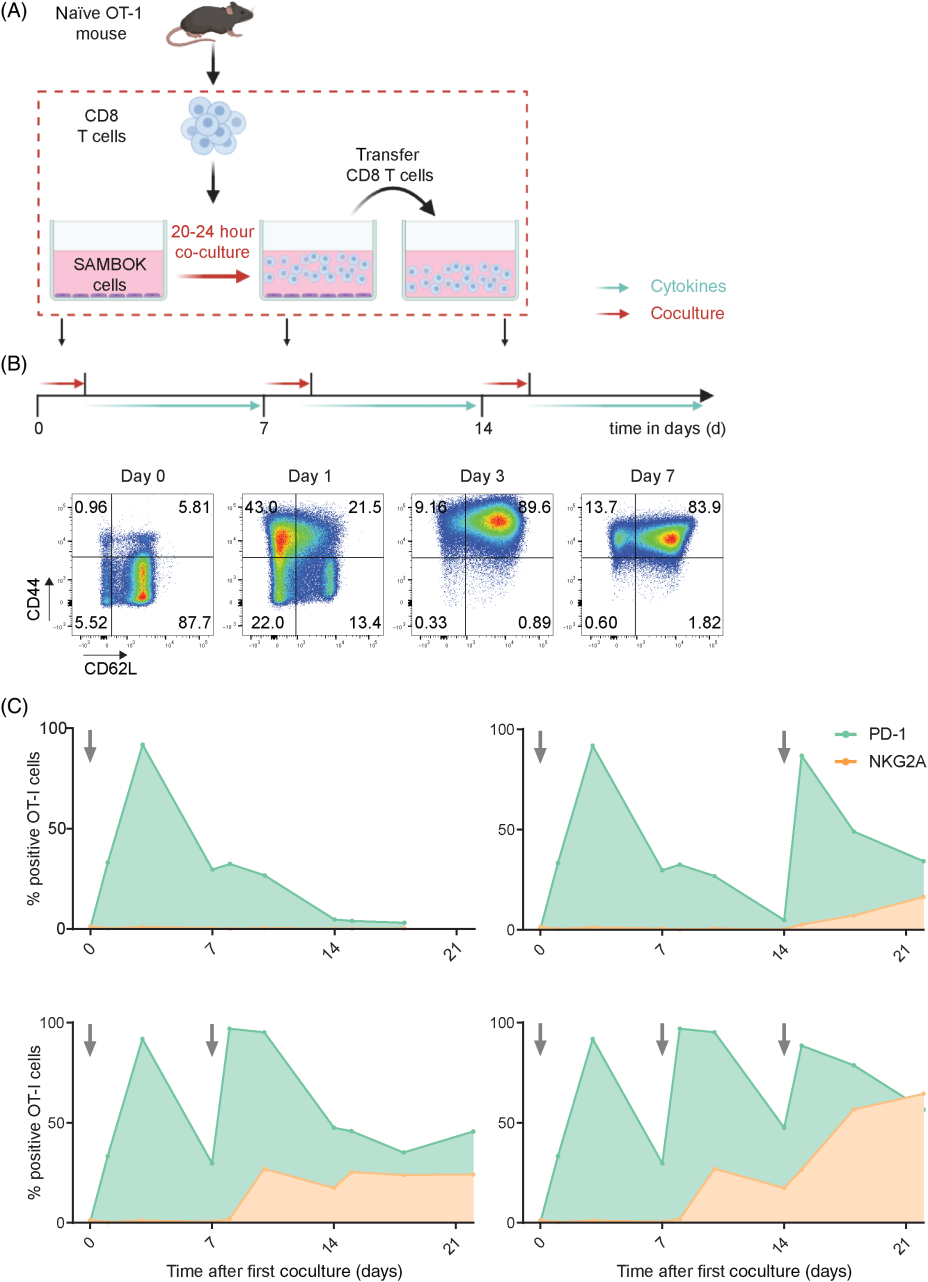
Synchronous activation of naïve OT‐I T cells demonstrates early induction of PD‐1, but late expression of NKG2A. (A) Schematic overview of this in vitro activation system for naïve CD8 T cells from OT‐I mice by coculture with adherent SAMBOK cells, fibroblasts transfected with B7‐1 (CD80) and the minigene encoding SigOVA_257‐264_. OT‐I T cells were overnight stimulated on these engineered presenting cells and then transferred to fresh wells with the homeostatic cytokine IL‐7, preventing chronic T cell receptor triggering. (B) Representative flow cytometry plots from three independent experiments displaying activation markers CD62L and CD44 on OT‐I T cells after the first stimulation by SAMBOK. (C) Surface expression of PD‐1 and NKG2A on OT‐I T cells after different stimulation schemes with SAMBOK at the indicated timepoints (arrow). One representative out of seven independent experiments is shown [Color figure can be viewed at wileyonlinelibrary.com]

We then employed a reductionist model in which naïve CD8 T cells were synchronously activated in vitro to test this hypothesis. Naïve OVA‐specific CD8 T cells were harvested from OT‐I mice and stimulated overnight by engineered fibroblasts expressing a minigene encoding the SIINFEKL epitope and the CD80 costimulatory molecule (SAMBOK cells)^35^ (Figure 2A). SAMBOK cells adhere to plastic and by transfer of CD8 T cells to clean wells after overnight stimulation, we achieved synchronized TCR/CD28 mediated activation of OT‐I T cells. These T cell cultures follow a weekly schedule of expansion and a resting phase as shown by the dynamic display of the CD44 activation marker and the regain of the CD62L differentiation marker at Day 7 (Figure 2B). PD‐1 expression was observed as early as 1 day after activation and peaked at Day 3 (Figure 2C), confirming that this inhibitory receptor is a marker of recent T cell activation in addition to marking exhaustion.^37^ PD‐1 was completely downregulated in the resting phase at Day 14. This was not due to selective cell death since hardly any cells died in this protocol. Repeated stimulations with SAMBOK cells resulted in a consistent pattern of quick PD‐1 upregulation followed by a steady decline during the resting phase (Figure 2C). In contrast, NKG2A expression was not detected at all after the first stimulation with SAMBOK cells, despite high levels of CD44 and PD‐1. Three weekly stimulations were required for maximal display of NKG2A by activated CD8 T cells and even then only approximately half of the OT‐I T cell population was positive. Prolongation of the resting phase hardly reduced frequencies of NKG2A‐positive OT‐I T cells. These findings were recapitulated with gp100‐specific TCR transgenic pmel T cells^38^ stimulated with peptide‐loaded matured dendritic cells (Figure S2). Some discrepancies with the OT‐I system could be explained by the less well‐controlled synchronization of the pmel T cell activation as witnessed by the heterogeneous expression of CD62L (Figure S2B). Together, these data demonstrated that these two inhibitory receptors are distinctly regulated: PD‐1 reflects recent TCR triggering and NKG2A relates to repeated sequential TCR triggering.

### 3.3 NKG2A and TIM‐3 are late checkpoints compared to immediate receptors PD‐1, LAG‐3 and TIGIT

**FIGURE 3:**
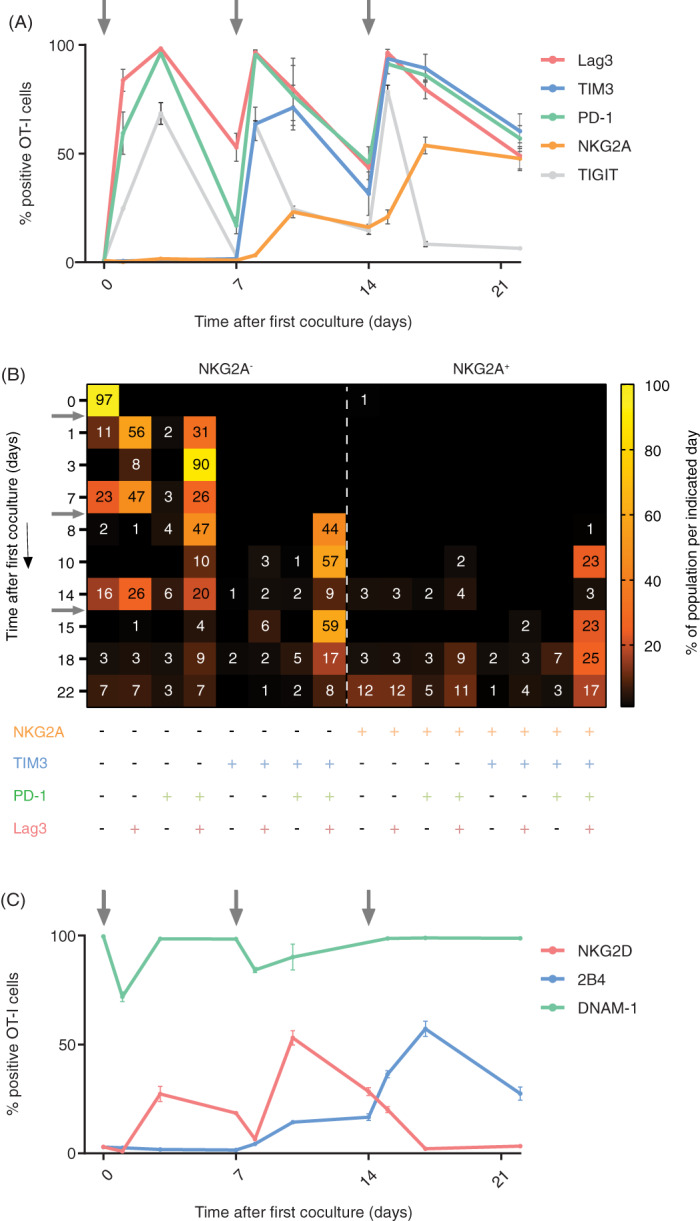
NKG2A expression in relation to other inhibiting and activating receptors. (A) Dynamic expression profiles of multiple inhibition receptors on OT‐I T cells after synchronous activation in the in vitro culture system, depicted in Figure 2A. Means and SD of triplicates from one representative out of five independent experiments are shown. (B) Heatmap of coexpression profiles of inhibition receptors from one representative experiment. Dynamics of 16 OT‐I populations based on Boolean gating. Shown are the percentages (only values >1 are depicted) of OT‐I T cells positive for the indicated receptors. Arrows indicate stimulations with SAMBOK cells. (C) Dynamic expression profiles of three activating receptors on OT‐I T cells from one representative out of three independent experiments. Data presented are means with SD of triplicates [Color figure can be viewed at wileyonlinelibrary.com]

Then, we examined expression of other inhibition and activating receptors in this synchronized system (Figure 3) The inhibition receptors LAG‐3 and TIGIT followed similar kinetics as PD‐1 (Figure 3A). Stimulations with SAMBOK led to a quick induction and steady decline in the resting phase. In contrast, TIM‐3 behaved like NKG2A, in that multiple sequential stimulations were required for expression. Coexpression profiling of these inhibitory receptors showed an early peak of PD‐1^+^LAG‐3^+^ OT‐I cells of up to 90% of the total population, which then started to coexpress TIM‐3 from the second stimulation onwards and finally NKG2A was induced after three consecutive stimulations with SAMBOK (Figure 3B). At the end of the experiment, the majority of OT‐I T cells had accumulated all analyzed inhibitory receptors. Analysis of activating receptors showed accumulating expression of NKG2D and 2B4 (CD244), but a decrease in DNAM‐1 (CD226) after TCR triggering (Figure 3C). Again, the pmel T cell system demonstrated similar results (Figure S2C). So, the induction of inhibitory receptors appears to be balanced by coexpression of activating receptors. The general picture arising from this culture system is an accumulating acquisition of inhibitory and activating receptors over time, of which NKG2A is the last receptor to be expressed.

### 3.4 TGF‐β enhances NKG2A expression, but is not required in vivo

**FIGURE 4:**
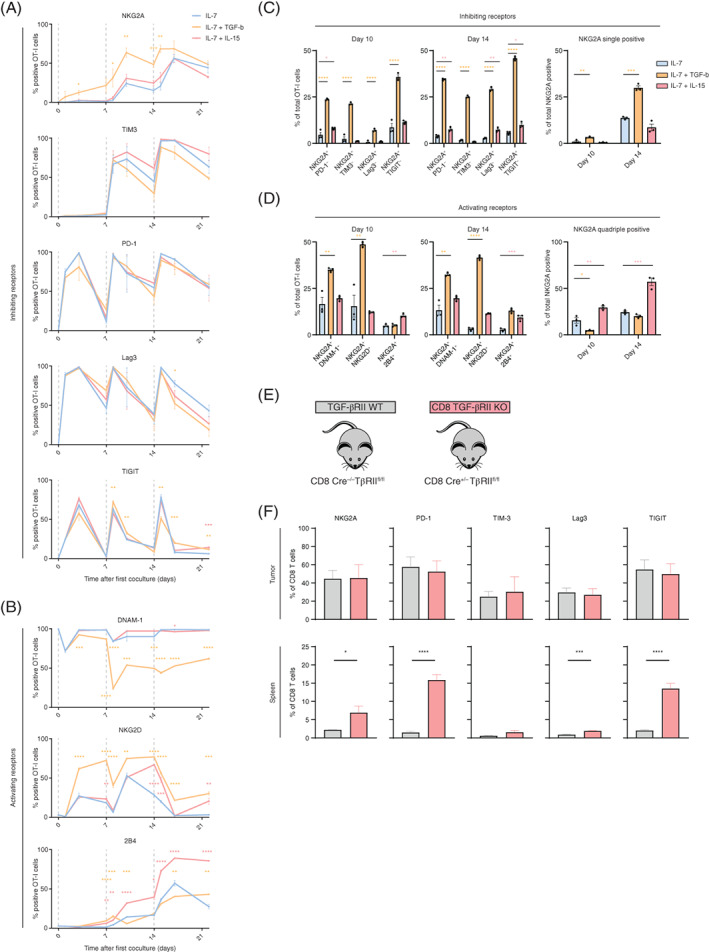
TGF‐β, but not IL‐15, enhances NKG2A expression on activated OT‐I T cells. OT‐I T cells were synchronously activated with SAMBOK cells, according to Figure 2A and then incubated in the presence of IL‐7 alone or in combination with 10 ng/mL IL‐15 or 5 ng/mL TGF‐β. (A‐B) Line graphs show the frequency of OT‐I T cells expressing (A) inhibiting receptors (n = 5) and (B) activating receptors (n = 3). Means and SEM of independent experiments are depicted. (C and D) Quantification of coexpression of (C) inhibiting receptors (n = 5) and (D) activating receptors (n = 3) in the several culture conditions. Data presented are means and SEM of independent experiments. One‐way ANOVA with Dunnett's multiple comparisons test was used for statistical analysis at the indicated time points. Color reflects cytokine condition compared to IL‐7 alone. (E and F) TGF‐βRII WT and CD8 TGF‐βRII KO (lacking the TGF‐β receptor II selectively in CD8 T cells) mice were inoculated with KPC3 tumors, which were 20 days later harvested together with spleens, dispersed and stained for flow cytometry. Data presented are means and SEM of four individual mice. Unpaired Student's *t* test for statistical analysis [Color figure can be viewed at wileyonlinelibrary.com]

As this reductionist system perfectly allows to integrate other signals regulating inhibitory receptor expression we included tumor‐microenvironment associated cytokines TGF‐β and IL‐15 in our culture system. NKG2A was strongly enhanced by addition of TGF‐β, whereas all other inhibitory receptors were hardly affected by this cytokine, including the other late onset receptor TIM‐3 (Figure 4A). The fraction of NKG2A^+^ OT‐I T cells doubled and its induction was already observed after one stimulation with SAMBOK in the presence of TGF‐β in a concentration dependent manner (Figure S3A). Addition of IL‐15 did not alter NKG2A expression, also not when complexed with the IL‐15R (Figures 4A and S3B). Furthermore, TGF‐β strongly reduced the expression of the activating receptor DNAM‐1, as previously reported for NK cells,^39^ but enhanced the expression of costimulatory molecule NKG2D (Figure 4B). Finally, IL‐15 induced 2B4 expression at later time points. These data show that TGF‐β augments the capacity of TCR triggering to induce NKG2A and NKG2D expression on CD8 T cells.

Coexpression profiling revealed NKG2A induction by TGF‐β on all subsets and that particularly TIM‐3^high^ and LAG‐3^high^ T cells coexpressed NKG2A (Figures 4C,D and S3C,D). Importantly, TGF‐β resulted in a larger population of NKG2A‐positive OT‐I T cells lacking other inhibitory receptors (Figure 4C). Simultaneously, TGF‐β induced a larger fraction of T cells coexpressing activating receptors DNAM‐1 and NKG2D (Figures 4D and S3E,F). At Day 10, nearly half of OT‐I cells were double positive for NKG2A and NKG2D. Together, these data show a potent induction of NKG2A by TGF‐β, but not by IL‐15, and a shift in the profile of immune regulatory receptors on CD8 T cells.

We then examined if TGF‐β is essential for expression of NKG2A in a mouse tumor model. The genetic KPC model for pancreatic ductal adenocarcinoma is known for production of TGF‐β^40^ and the KPC3 line was grown in conditional knockout mice for the TGF‐β receptor II.^33^ This floxed gene was bred unto CD8‐driven Cre^+/−^ background, resulting in mice that selectively lost this essential TGF‐β receptor in CD8 T cells (Figures 4E and S3G‐I). No major differences were observed in lymphocyte frequencies within tumors or spleens (Figure S3G) and also the differentiation status of CD8 TIL, based on CD44 and CD62L, was comparable between CD8‐Cre^+/−^ and CD8‐Cre^−/−^ mice (Figure S3H). Importantly, we did not observe lower frequencies of tumor‐infiltrating NKG2A^+^ CD8 T cells in CD8‐Cre^+/−^ TβRII^fl/fl^ mice, indicating that TGF‐β signaling is not essential for NKG2A induction on TIL in vivo (Figure 4F). Moreover, differences were neither found for other inhibitory receptors on CD8 TIL. In the spleen and blood of knockout mice even higher fractions of CD8 T cells with inhibitory receptors were detected (Figures 4F and S3H); however, this was due to a general increase of CD8 effector cell frequencies (CD44^+^CD62L^−^KLRG1^+^). Loss of the TGF‐β receptor on CD8 T cells apparently led to more CD8 T cell activation due to absence of immunosuppressive TGF‐β triggering. Indeed, inhibitory receptors on other lymphocyte lineages were not affected (Figure S3I).^41^ We concluded that TGF‐β signaling is not crucial, but can enhance the expression of NKG2A on CD8 T cells in vitro.

### 3.5 Induction of NKG2A is related to actively dividing cells

**FIGURE 5:**
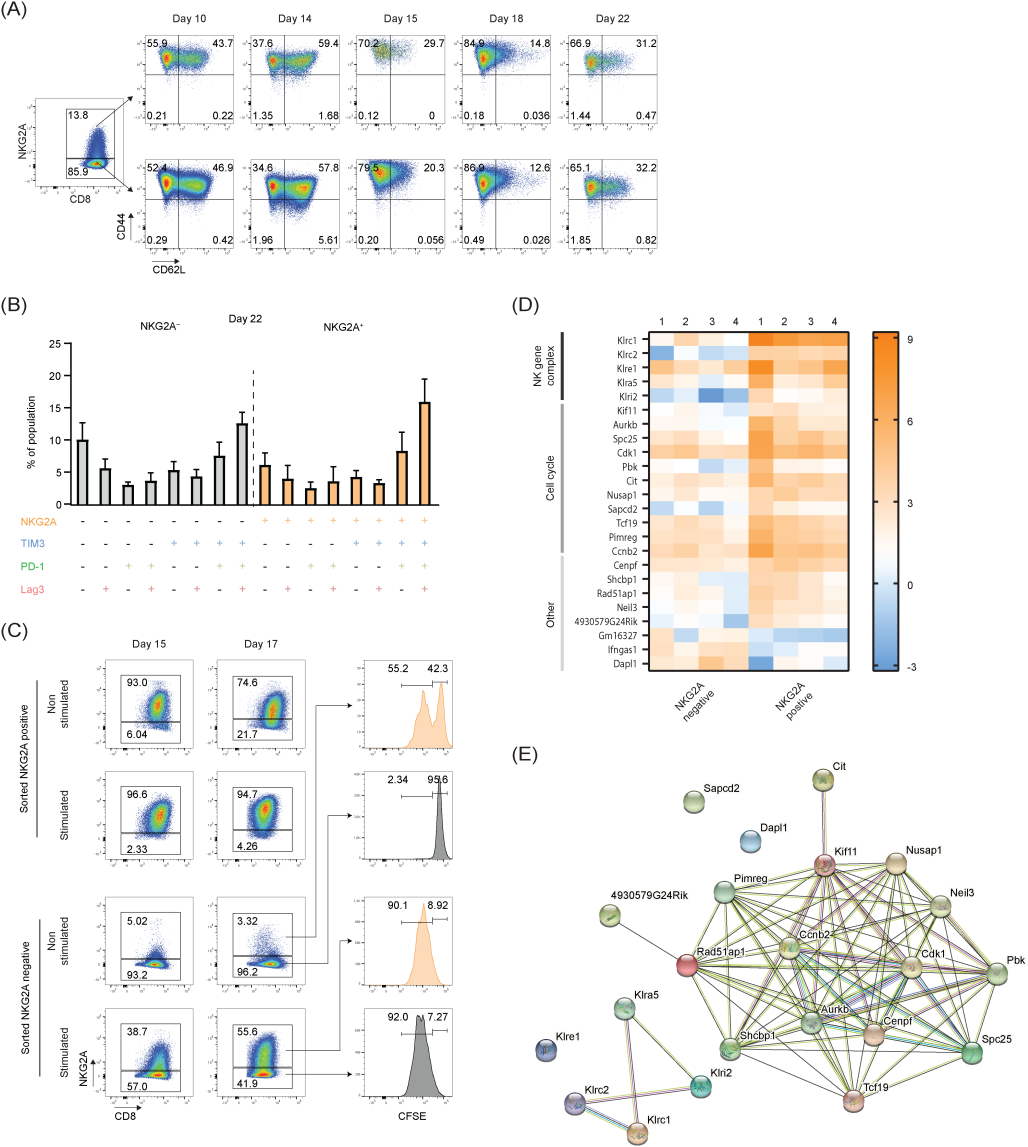
NKG2A expression is associated with cell division. (A) Representative flow cytometry plots from three independent experiments showing CD44 and CD62L expression by NKG2A^+^ and NKG2A^−^ OT‐I T cells. (B) Frequencies of OT‐I T cell populations with unique coexpression profiles of NKG2A, TIM‐3, PD‐1 and LAG‐3 at Day 22. Frequencies of 16 separate OT‐I T cell populations expressing distinct combinations of receptors are depicted. Populations expressing NKG2A are indicated in orange. Frequencies are means and SEM from five independent experiments. (C) OT‐I T cells were isolated at Day 14 before the third coculture with SAMBOK cells and FACS sorted based on their NKG2A expression (Figure S3B). NKG2A^+^ and NKG2A^−^ OT‐I populations were split, labeled with cell tracer CFSE and subsequently cultured in medium alone (nonstimulated) or in the presence of SAMBOK cells (stimulated). Their NKG2A expression was measured at the indicated time points. This experiment was repeated with similar results. (D) Heatmap of statistically significant differentially expressed genes (DEG) comparing NKG2A^+^ and NKG2A^−^ OT‐I T cells directly after FACS sorting. Depicted are all 24 DEGs between these two populations. Shown are expression data from cocultures of four individual mice. (E) STRING analysis of 21 upregulated proteins and three downregulated proteins in NKG2A‐positive OT‐I T cells [Color figure can be viewed at wileyonlinelibrary.com]

Though the percentage of NKG2A OT‐I cells in our culture system approached 70 % when stimulated in the presence of TGF‐β, still a part of the synchronously stimulated OT‐I population remained negative. We systematically investigated intrinsic differences coinciding with NKG2A expression (Figure 5). First, NKG2A expression was not related to general T cell differentiation, as CD44 and CD62L were evenly distributed among NKG2A^+^ and NKG2A^−^ OT‐I T cells (Figure 5A). Second, we analyzed the coexpression of other inhibitory receptors on the NKG2A^+^ vs NKG2A^−^ OT‐I populations (Figures 5B and S4A). At Day 22 of coculture, very similar percentages of PD‐1, LAG‐3 and TIM‐3 were found and Boolean gating of receptor combinations also revealed even distributions. Third, we examined if NKG2A^−^ cells were intrinsically resistant to express the gene or that the increase over time was the result of selective outgrowth of the NKG2A^+^ population (Figure 5C). OT‐I T cells were sorted by FACS on basis of NKG2A expression at Day 14 just before the third stimulation (Figure S4B) and the cells were rested or activated with SAMBOK. More than half of the NKG2A^−^ T cells induced NKG2A after SAMBOK stimulation, indicating that the receptor is actively switched on in these cells after repeated TCR triggering (Figure 5C). Interestingly, a small percentage of cells induced NKG2A even without extra stimulation and thus represented late onset expression. We hypothesized that these cells might still have an activated status amidst resting OT‐I cells. Sorted NKG2A^−^ cells were labeled with cell tracer CFSE to monitor division and indeed, half of these late onset T cells had divided between Day 14 and 17, whereas T cells that remained negative for NKG2A had not divided (Figure 5C). These data indicate that NKG2A expression is associated with cell division and thus was expressed by activated T cells.

Finally, we performed transcriptome analysis to identify all possible differences between NKG2A^+^ and NKG2A^−^ OT‐I cells at Day 21. Transcriptomes were compared and, strikingly, only 24 genes were differentially expressed, demonstrating the great similarity of these two OT‐I populations (Figure 5D). In addition to the *KLRC1* gene that encodes NKG2A, four other C‐type lectin receptors were found in NKG2A‐positive OT‐I T cells: *KLRC2* (coding NKG2C), *KLRi2*, *KLRe1* and *KLRa5*. All these receptors are encoded in close proximity of the same natural killer gene complex (NKC) of mouse chromosome 6 of *KLRC1*, suggesting coordinated transcription of this small cluster. Interestingly, 11 out of the 24 differential genes were involved in mitotic cell cycle (GO term 0007049) and were significantly higher expressed in NKG2A‐positive cells (Figure 5D). The function of these 11 gene products is tightly linked, as illustrated by STRING analysis (Figure 5E) and they are involved in the process of mitosis and, more specifically, the mitotic spindle formation and segregation of sister chromatids (GO term 0005819). This transcriptome analysis confirmed our flow cytometry data and implied that NKG2A expression is related to actively dividing cells. Together, these data demonstrate that NKG2A is a hallmark of activated, dividing CD8 T cells. Moreover, the data infer that NKG2A induction is independent from other well‐known checkpoint receptors, including PD‐1, LAG‐3, TIGIT, TIM‐3 and activating receptors DNAM‐1, NKG2D and 2B4.

### 3.6 NKG2A marks a unique population of CD8 TILs

**FIGURE 6:**
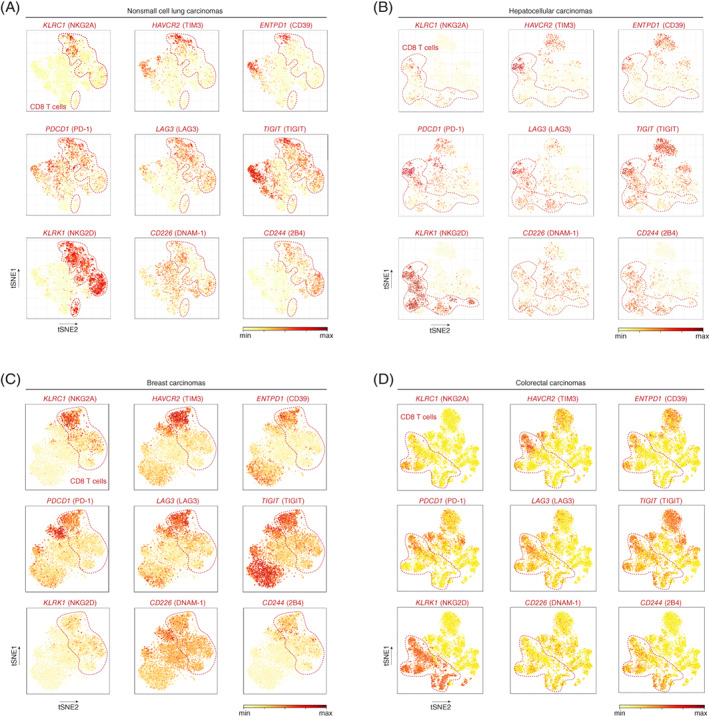
Cluster analysis and expression levels of single cell transcriptomes of (A) 14 nonsmall cell lung carcinomas^29^ (9055T cells from blood and tumor), (B) 5 hepatocellular carcinomas^30^ (4070T cells from blood and tumor), (C) 2 breast carcinomas^32^ (5759T cells from blood and tumor) and (D) 12 colorectal carcinomas^31^ (11138T cells from peripheral blood, adjacent normal and tumor tissues) [Color figure can be viewed at wileyonlinelibrary.com]

We were interested to know how these insights relate to the clinical situation, and therefore examined NKG2A expression together with other immune checkpoints by CD8 T cells in various human tumor types, exploiting open‐source databases of single‐cell transcriptome profiles. Single‐cell transcriptomes of T cells from nonsmall‐cell lung carcinomas,^29^ hepatocellular carcinomas,^30^ colorectal carcinoma^31^ and breast carcinoma^32^ revealed that NKG2A (*KLRC1*), TIM‐3 (*HAVCR*2) and CD39 (*ENTPD1*) were expressed in the same unique CD8 T cell clusters and that PD‐1 (*PDCD1*), LAG‐3 (*LAG3*), TIGIT (*TIGIT*), NKG2D (*klrk1*), DNAM‐1 (*CD226*) and 2B4 (*CD244*) are far more globally distributed among multiple CD8 T cell clusters (Figure 6). Subtle differences in expression patterns were observed between the tumor types and are likely caused by cell context‐dependent differences in tumor‐induced immune landscapes. In line with our mouse studies, human CD8 TILs that are positive for NKG2A also frequently coexpressed most of the other analyzed coinhibitory receptors, suggesting that NKG2A marks a population of repeatedly activated TILs.

**FIGURE 7:**
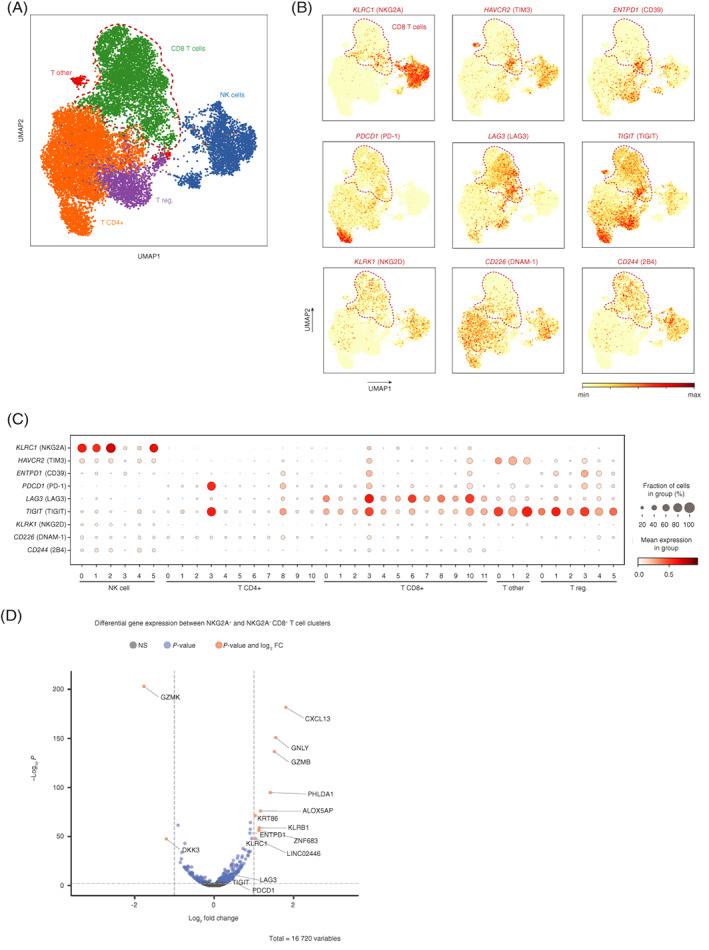
Restricted distribution of NKG2A within CD8 TIL clusters in head and neck carcinoma. (A) Two‐dimensional UMAP plots displaying single cell transcriptomes of 14242T cells and 2820 NK cells from 13 untreated oropharyngeal squamous cell carcinomas. Each dot represents a single cell and red dotted line denote CD8 TIL. (B) Expression levels of surface immune receptors are depicted in color code. (C) Clusters of TIL were analyzed for transcript levels of indicated receptors. (D) Volcano plot of differentially expressed genes on CD8 TIL clusters 3 and 10 compared to all other CD8 TIL clusters. Shown are Benjamini‐Hochberg adjusted *P*‐values [Color figure can be viewed at wileyonlinelibrary.com]

In addition to these databases, the single‐cell transcriptomes of 14 242 intratumoral T cells and 2820 NK cells were determined from 13 untreated oropharyngeal squamous cell carcinomas (OPSCC).^21^ A two‐dimensional UMAP plot displayed good separation of CD8 T cells, CD4 T cells, regulatory T cells and NK cells (Figures 7A and S5). NKG2A, TIM‐3 and CD39 were expressed in a limited number of clusters compared to the more globally distributed receptors PD‐1, LAG‐3, TIGIT, NKG2D, DNAM‐1 and 2B4 (Figure 7B,C). NKG2A was expressed on all NK cell clusters. The activating receptors NKG2D (*klrk1*), DNAM‐1 (*CD226*) and 2B4 (*CD244*) were expressed by most NK cells and also most CD8 T cells (NKG2D and 2B4) or CD4 T cells (DNAM‐1). In line with the other tumor types, the NKG2A‐positive CD8 T cell was highly enriched for most other analyzed checkpoints (Figure 7B‐C). Vice versa, the globally distributed receptors were evenly expressed in NKG2A‐negative vs NKG2A‐positive clusters. Moreover, CD39 and TIM‐3 were enriched on NKG2A‐expressing CD8 TIL Clusters 3 and 10 (Figure 7D). Finally, we performed a differentially expressed gene analysis on NKG2A^+^ CD8 T cells (Clusters 3 and 10) compared to NKG2A^−^ CD8 T cells (all other clusters) and identified *CXCL13*, *granulysin*, *granzyme B* and *KLRB1*, suggesting a cytolytic phenotype. Together, our results imply that NKG2A is, similar to TIM‐3 and CD39, a late immune checkpoint receptor on CD8 T cells induced after repeated cognate antigen interactions and marks chronically activated cells with potential cytolytic function.

## 4 DISCUSSION

Our study revealed that NKG2A is, like TIM‐3 and CD39, a late inhibitory receptor for CD8 T cells that is induced after repeated TCR‐mediated stimulations. In contrast, inhibitory receptors PD‐1, LAG‐3 and TIGIT reflect recently activated CD8 T cells and these molecules are more transiently expressed. Interestingly, these early receptors were rather universally distributed among CD8 T cell clusters in the tumor microenvironment of human nonsmall cell lung carcinoma, colon carcinoma, hepatocellular carcinoma, oropharyngeal carcinoma and breast cancer tumors, whereas the typical the late receptors NKG2A, TIM‐3 and CD39 were selectively expressed by a limited number of CD8 T cell clusters.

NKG2A expression was furthermore associated with dividing CD8 T cells, suggesting that this particular TIL population represents a chronically activated subset of CD8 T cells in tumors and potentially marks the tumor‐reactive fraction, as was suggested for PD‐1^high^, TIM‐3 and CD39 positive T cells.^42^, ^43^, ^44^, ^45^ The CD39 molecule is a surface ectonucleotidase involved in the creation of an antiinflammatory microenvironment and has been described as a marker for tumor‐specific CD4 and CD8 TILs.^21^, ^43^, ^44^ Our study suggests that NKG2A is expressed by the same TIL subsets and may discriminate tumor‐specific CD8 T cells from bystander TILs. The coexpression of NKG2A and TIM‐3 could also be explained by the indicated association of TIM‐3 as a receptor expressed on IFN‐γ‐producing CD4 and CD8 T cells^46^ with high transcription factor T‐bet^7^ and nuclear factor interleukin 3 (NFIL3).^47^ Our transcriptome analysis revealed a cytolytic phenotype for the CD8 T cell clusters expressing NKG2A.

In contrast to most other inhibitory receptors NKG2A is selectively expressed by cytolytic lymphocytes, such as NK cells and CD8 T cells. Targeting this checkpoint with blocking antibodies thus selectively enables these antitumor lymphocytes without unwanted activation of T_regs_ or disbalancing helper T cells.^48^, ^49^ The induction of NKG2A on NK cells is well defined, showing constitutive expression on educated cells and losing its expression during differentiation. Transcription factor GATA‐3 is important for its gene expression.^50^, ^51^, ^52^ In contrast, NKG2A is not expressed on mature naïve CD8 T cells but is induced upon TCR activation in immune reactive tumor microenvironment (TME), like after therapeutic peptide vaccination.^18^, ^53^ This has been assigned to an IL‐2 induced, calcineurin‐dependent suppression of NKG2A expression on naïve CD8 T cells.^54^ We demonstrated that the regulation of NKG2A expression and induction on CD8 T cells is dependent not simply on cognate antigen interactions, but on repeated TCR stimulation and is related to an actively dividing phenotype. This also fits with the findings that rapidly dividing cells in the TME express more inhibitory receptors^55^ and the observation that NKG2A expression is associated with proliferative potential on virus‐specific CD8 T cells.^56^ In addition, we confirmed earlier reports on enhanced expression of NKG2A on CD8 T cells by TGF‐β.^20^, ^53^, ^57^ Importantly, TGF‐β is generally present in the TME.^58^, ^59^ However, we here also show that TGF‐β signaling in CD8 T cells is not a prerequisite for NKG2A expression in vivo, as similar frequencies of NKG2A^+^ CD8 T cells were found in the TME of pancreatic mouse carcinomas in TGF‐β receptor II deficient hosts compared to wild type mice. Interestingly, our CD8‐specific knockout model also showed similar percentages of PD‐1 expressing CD8 T cells, whereas a previous publication demonstrated that PD‐1 was enhanced by TGF‐β1 and SMAD3.^60^ This difference might be explained by the pleiotropic effects of TGF‐β in mammals and the rich consequences of TGF‐β signaling, such as dampening CD8 T cell proliferation.^41^ In other preclinical models in which TGF‐β signaling was hampered in CD8 T cells, no effect on the expression of NKG2A was observed.^41^

We previously profiled CD8 TIL of cervical carcinomas by mass cytometry (CyTOF) and revealed a strong coexpression of NKG2A CD8 T cells with the E‐cadherin binding integrin CD103, indicating that NKG2A might be enriched on tissue‐resident memory T cells (T_RM_).^18^ T_RM_ cells are generated by T cell priming, but do not recirculate like effector memory T cells or central memory T cells. T_RM_ are retained in tissues via retention integrins CD49a, CD49b, CD103 and the CD69/S1PR system, a program that is regulated by the transcription factors BLIMP and Hobit.^61^, ^62^, ^63^ Interestingly, our current single cell transcriptome analysis of CD8 T cells from oropharyngeal carcinomas revealed the T_RM_ master regulator Hobit (*ZNF683* gene) as differentially expressed gene in NKG2A positive T cell clusters. Enriched NKG2A expression in the T_RM_ differentiation pathway was also found in lung tissue^61^ and in CD8 T cells infiltrating triple‐negative breast cancer^32^ and nonsmall cell lung carcinoma.^29^ The significant association between T_RM_ gene signature and improved survival of cancer is obviously of great importance and suggests that blockade of NKG2A might unleash the potency of CD8 T_RM_ cells. Future investigations need to delineate the origins of T_RM_ in cancer and their inhibitory receptor profile.

## CONFLICT OF INTEREST

Thorbald van Hall and Sjoerd H. van der Burg are recipients of a research grant from Innate Pharma. The remaining authors declare no conflict of interest.

## ETHICS STATEMENT

The head and neck carcinoma samples were derived in context of study P07.112 that was approved by the local medical ethical committee of Leiden University Medical Center and in agreement with the Dutch law. Blood and tumor tissue were collected and used according to the Dutch Federal of Medical Research Association guidelines. All patients received standard‐of‐care treatment and provided written informed consent. All mouse experiments were controlled by the animal welfare committee (IvD) of the Leiden University Medical Center or Erasmus University Medical Center (Rotterdam) and approved by the national central committee of animal experiments (CCD) under the permit numbers AVD116002015271 and AVD101002017867, in accordance with the Dutch Act on Animal Experimentation and EU Directive 2010/63/EU.

## Supporting information


**Appendix S1**: Supplementary Information

## Data Availability

The single cell RNA‐seq data from human head and neck carcinomas generated in our study are available at https://icbi-lab.github.io/borst2021. The RNAseq data generated in our study are available under identifier PRJEB47938 or ERP132259 at https://www.ebi.ac.uk/ena/browser/submit. Other data that support the findings of our study are available from the corresponding author upon request.
