# Supplementary material for: NKG2A is a late immune checkpoint on CD8 T cells and marks repeated stimulation and cell division
Source: Int J Cancer. 2021 Nov 10;150(4):688–704. doi: 10.1002/ijc.33859 (PMC9299709; doi:10.1002/ijc.33859)
Supplement: Supplementary file 1 — Appendix S1: Supplementary Information [file IJC-150-688-s001.pdf]

NKG2A is a late immune checkpoint on CD8 T cells and marks repeated stimulation and cell division

Linda Borst, Marjolein Sluijter, Gregor Sturm, Pornpimol Charoentong, Saskia J. Santegoets, Mandy van Gulijk, Marit J. van Elsas, Christianne Groeneveldt, Nadine van Montfoort, Francesca Finotello, Zlatko Trajanoski, Szymon M. Kiełbasa, Sjoerd H. van der Burg and Thorbald van Hall

Table of contents:

Supplementary Table S1

Supplementary Table S2

Supplementary Figure S1

Supplementary Figure S2

Supplementary Figure S3

Supplementary Figure S4

Supplementary Figure S5

Table S1: quality of single cell RNA sequencing (scRNA-seq) of human head/neck carcinoma samples

Sample ID	Total number of sequenced reads per sample [a]	Total number of uniquely mapped reads per sample[b]	Total number of called cells[c]	Median number (and range) of uniquely mapped reads per called cell[c]	Median rRNA rate (and range) per called cell[c,d]	Median number (and range) of detected genes per called cell[c]
H68	145639312	12920163	2,801	2,846 (1,237 – 45,037)	0.24 (0.01 - 0.45)	1,490 (740 – 6,241)
H188	228929000	190873981	2,063	2,654 (1,206 – 38,909)	0.25 (0.01 - 0.43)	1,421 (727 – 4,787)
H208	465578498	388028467	2,284	3,498 (1,154 – 64,179)	0.21 (0.02 - 0.45)	1,753 (766 – 6,586)
H185	197694189	173702555	1,912	2,608 (1,237 – 42,851)	0.19 (0.02 - 0.45)	1,458 (723 – 5,294)
H197	267169605	239072927	1,908	2,908 (1,317 – 57,924)	0.11 (0.00 - 0.34)	1,500 (736 – 7,681)
H176	253092503	219588089	1,611	2,725 (1,217 – 51,914)	0.21 (0.01 - 0.39)	1,528 (736 – 5,502)
H160	220886238	196941914	3,191	2,423 (1,222 – 60,439)	0.24 (0.02 - 0.38)	1,337 (709 – 7,301)
H149	357505855	310974775	1,131	2,472 (1,286 – 33,515)	0.26 (0.02 - 0.44)	1,377 (787 – 4,907)
H211	356305736	308742322	2,930	2,192 (993 – 77,325)	0.31 (0.02 - 0.51)	1,245 (704 – 7,324)
H141	602938326	487000938	2,966	2,362 (1,041 – 58,100)	0.32 (0.03 - 0.54)	1,304 (708 – 7,088)
H182	286349621	252008749	2,561	2,587 (1,165 – 43,770)	0.24 (0.01 - 0.43)	1,408 (727 – 6,566)
H205	385387851	341106543	1,178	2,940 (1,158 – 63,142)	0.18 (0.02 - 0.41)	1,521 (702 – 7,983)
H143	281522477	243022789	1,760	2,560 (1,184 – 55,511)	0.23 (0.01 - 0.38)	1,450 (737 – 7,160)

[a] Raw files were processed with cellranger 3.0

[b] Reference genome GRCh38

[c] After quality control and filtering for e.g., possible cell doublets, potential apoptotic cells.

[d] rRNA rate = Ratio of all reads aligned to rRNA regions to total uniquely mapped reads. Range is defined as the minimum – maximum (i.e., median (minimum – maximum)).

Table S2: bulk RNA sequencing quality of cultured OT-1 T cells

Sample ID	Total number of sequenced reads	Total number of uniquely mapped reads[a]	Ratio of all reads aligned to rRNA regions to total uniquely mapped reads (rRNA rate)	Ratio of exon-mapped reads to total uniquely mapped reads (Expression Profile Efficiency)	Total number of detected transcripts with reads ≥1
OT-1 NKG2A- no1	67609138	42027032	0.104	0.971	17890
OT-1 NKG2A- no2	74461605	44992992	0.119	0.944	22459
OT-1 NKG2A- no3	84259094	54150124	0.085	0.93	25944
OT-1 NKG2A- no4	62477383	41116967	0.119	0.961	19048
OT-1 NKG2A- no5	76454355	49739174	0.1	0.946	20414
OT-1 NKG2A+ no1	24379493	16874847	0.039	0.947	18029
OT-1 NKG2A+ no2	37249452	23749289	0.116	0.967	17996
OT-1 NKG2A+ no3	69913322	43623956	0.057	0.956	22061
OT-1 NKG2A+ no4	72245203	45774380	0.099	0.951	21071
OT-1 NKG2A+ no5	69778037	44215881	0.098	0.911	24460

[a] reference genome GRCm38.96 was used.

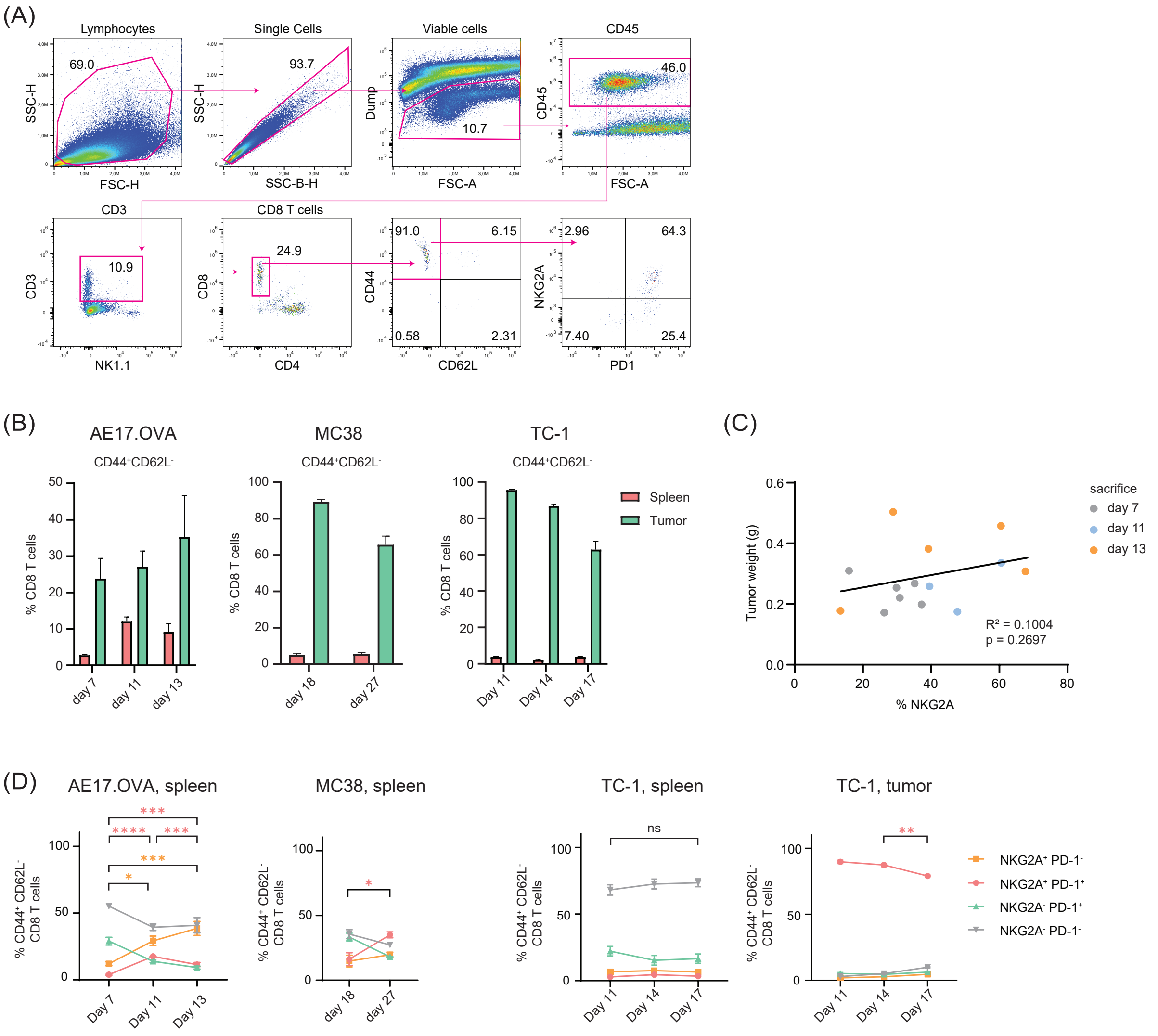

Supplementary Figure 1. In vivo expression of NKG2A and PD-1 on activated CD8 T cells. (A) Flow cytometry gating strategy for intratumoral and splenic CD8 T cells. (B) Proportion of activated ($CD44^{+}CD62L^{-}$) CD8 T cells out of all CD8 T cells in spleen and tumor from AE17.OVA, MC38 and TC-1 tumor-bearing animals. (C) Correlation plot of tumor weight and frequency of NKG2A-expressing CD8 T cells from AE17.OVA tumor samples. (D) Frequencies of CD8 T cells expressing NKG2A and/or PD-1 from spleens of AE17.OVA, MC38 and TC-1 and in TC-1 tumors. Data are derived from minimal 6 different mice and represent mean with SEM. Four subsets were plotted over time. One-way ANOVA with Holm-Sidak's multiple comparisons test was used for statistical analysis.

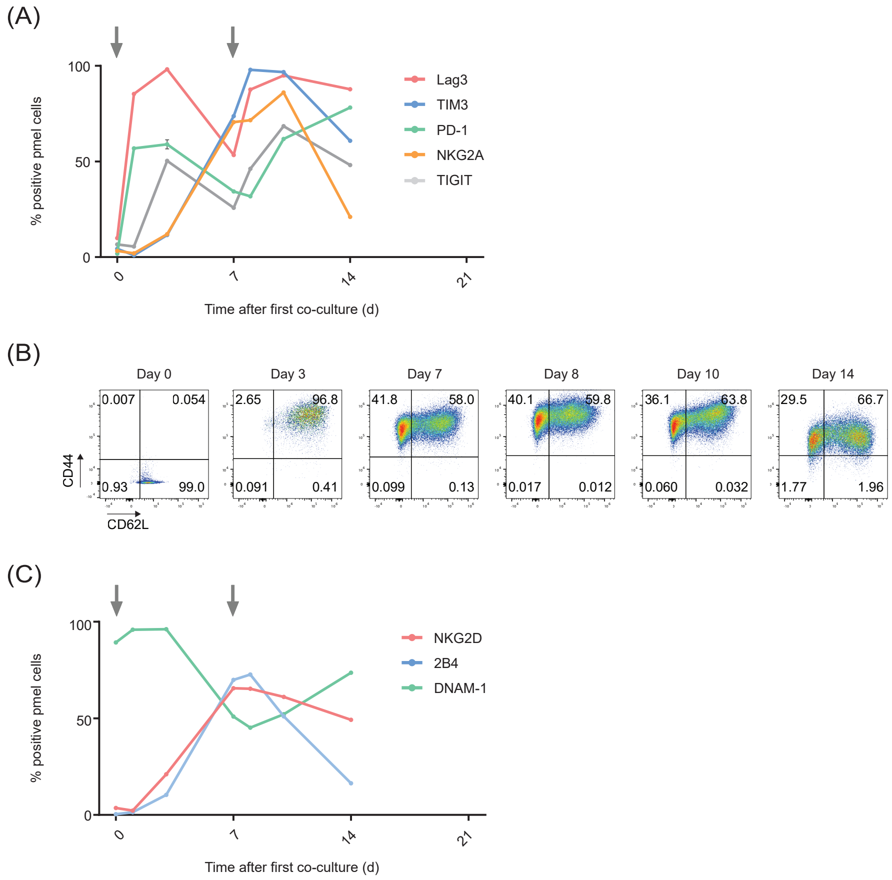

Supplementary Figure 2. Stimulation of naïve TCR-transgenic pmel CD8 T cells recognizing the gp100 peptide in H-2D^{b} with peptide-pulsed dendritic cell line D1. (A) Frequencies of pmel T cells expressing the indicated inhibitory receptors over time. (B) Flow cytometry dotplots of activation markers CD44 and CD62L, showing a diverse population of pmel T cells. (C) Frequencies of pmel T cells expressing the indicated activating receptors over time.

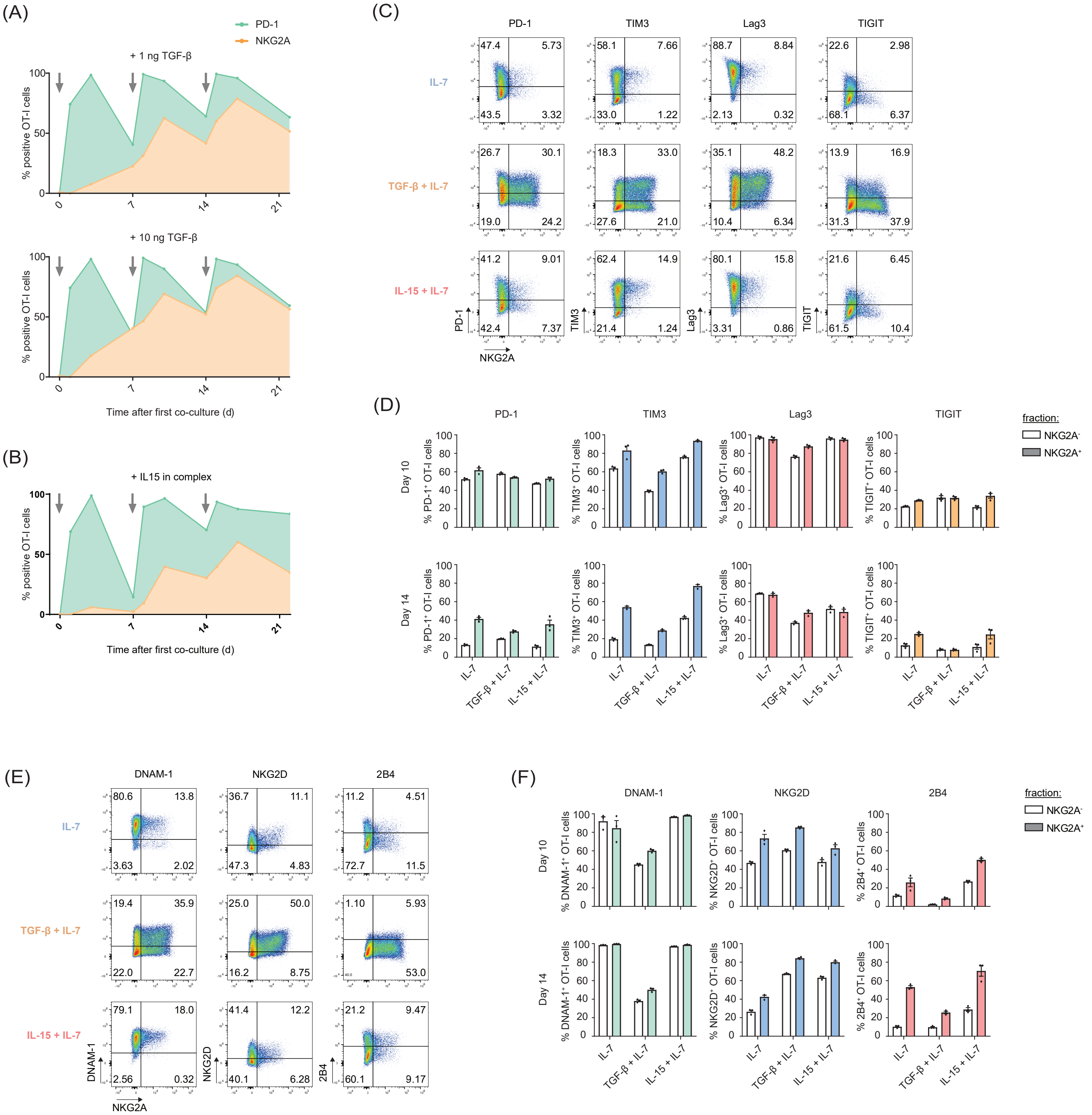

Supplementary Figure 3. (A) Percentages OT-I T cells displaying PD-1 and NKG2A in the presence of IL-7 and 1 ng/ml or 10 ng/ml TGF-β. Arrows indicate OT-I stimulation with antigen expressing SAMBOK cells. (B) Percentages OT-I T cells displaying PD-1 and NKG2A in the presence of IL-7 and 50 ng/ml IL-15 with 100 ng/ml IL15-Rα-Fc in complex. (C-D) Flow cytometric plots (C) and quantification (D) of co-expression of inhibitory receptors on OT-I T cells incubated with cytokines TGF-β or IL-15. (E-F) Flow cytometric plots (E) and quantification (F) of co-expression of activating receptors on OT-I T cells incubated with cytokines TGF-β or IL-15. Means and SEM of 3-5 independent experiments. Representative flow cytometry plots are from day 10.

(G)

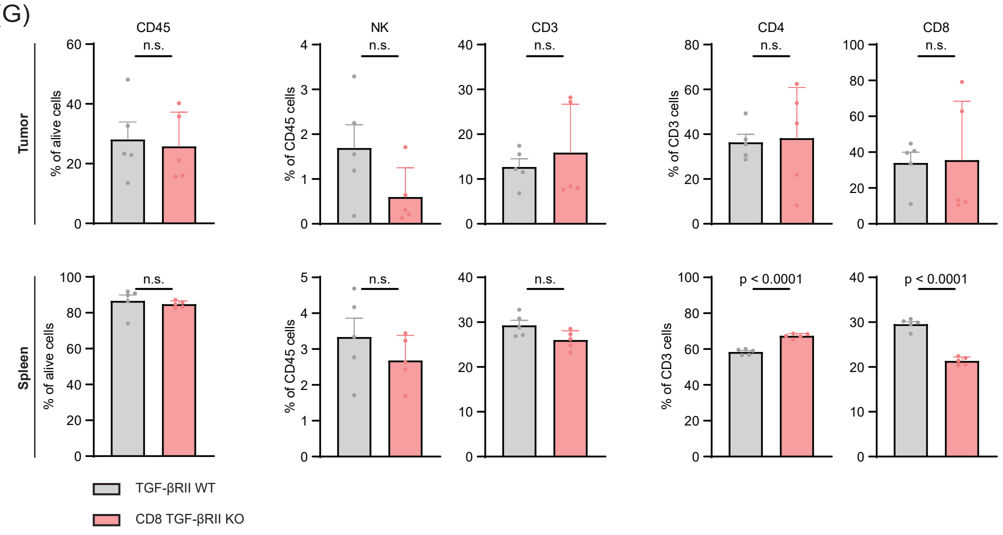

(H)

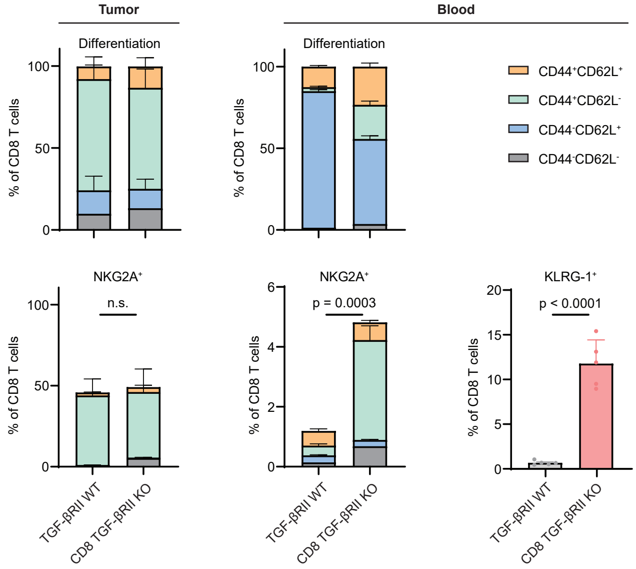

(I)

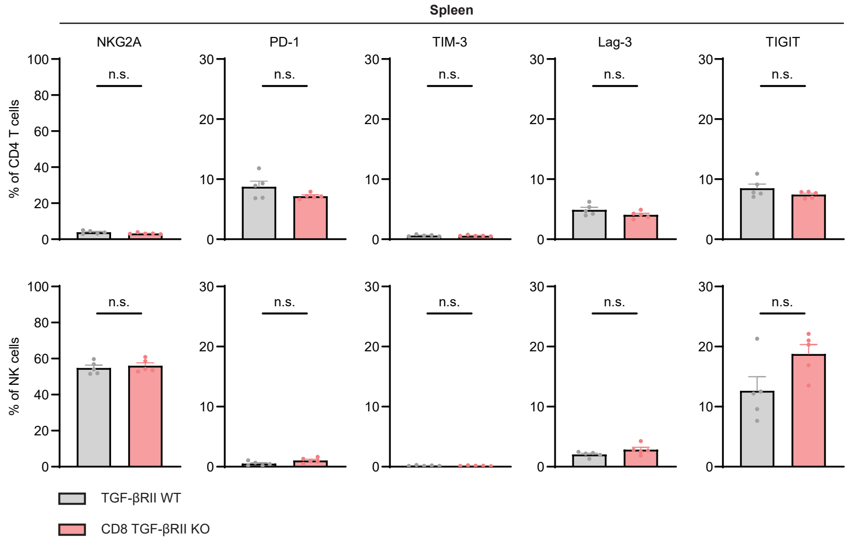

Supplementary Figure 3. (G-I) TGF-βRII WT and CD8 TGF-βRII KO (lacking the TGF-β receptor II selectively in CD8 T cells) mice were inoculated with KPC3 tumors. 20 days after tumor inoculation tumor, spleen and blood were harvested, dispersed and stained for flow cytometry to analyse (G) lymphocytes infiltrate, (H) differentiation state and the NKG2A expression visualized per differentiated population, (I) expression of inhibiting receptors. Data presented are means ± SEM. Unpaired Student's t test was used for statistical analysis.

(A)

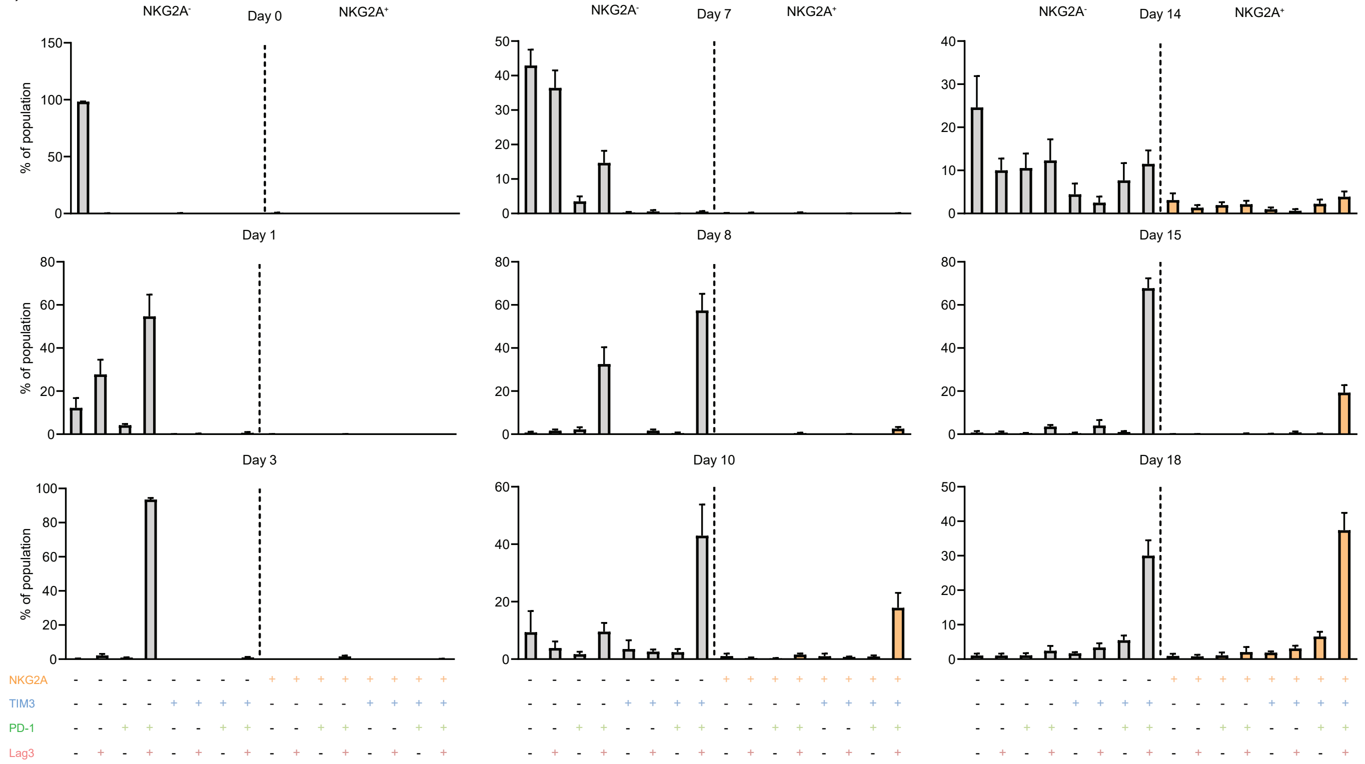

(B)

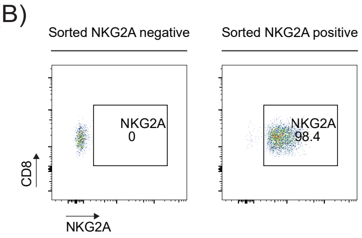

Supplementary Figure 4. (A) Co-expression of several inhibitory receptors on *in vitro* cultured OT-I T cells at different time points. (B) Samples from OT-I T cells from day 21 directly after FACS sorting based on NKG2A.

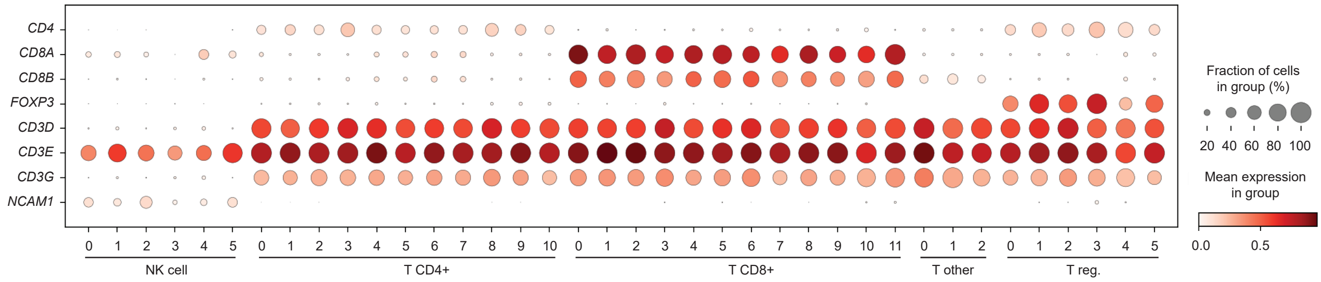

Supplementary Figure 5. Transcript levels of lineage markers in all clusters from single-cell transcriptomics of T cells and NK cells from oropharyngeal squamous cell carcinomas from Fig. 7.
